# Prognostic Significance of CD105- and CD31-Assessed Microvessel Density in Paired Biopsies and Surgical Samples of Laryngeal Carcinoma

**DOI:** 10.3390/cancers12082059

**Published:** 2020-07-25

**Authors:** Gino Marioni, Leonardo Franz, Giancarlo Ottaviano, Giacomo Contro, Giulia Tealdo, Alessandro Carli, Anna Chiara Frigo, Piero Nicolai, Lara Alessandrini

**Affiliations:** 1 Department of Neuroscience-DNS, Otolaryngology Section, University of Padova, 35128 Padova, Italy; leonardo.franz1991@yahoo.it (L.F.); giancarlo.ottaviano@unipd.it (G.O.); giacomo.contro@gmail.com (G.C.); giulia.tealdo@gmail.com (G.T.); alessandro.carli.1@studenti.unipd.it (A.C.); piero.nicolai@unipd.it (P.N.); 2 Department of Cardiac-Thoracic-Vascular Sciences and Public Health, Padova University, 35128 Padova, Italy; annachiara.frigo@unipd.it; 3 Department of Medicine-DIMED, University of Padova, 35128 Padova, Italy; lara.alessandrini@aopd.veneto.it

**Keywords:** laryngeal squamous cell carcinoma, angiogenesis, CD105, CD31, biopsy, surgical specimen, prognosis

## Abstract

Small pretreatment laryngeal biopsies may not fully represent a tumor’s biological profile. This study on laryngeal squamous cell carcinoma (LSCC) aimed to investigate the prognostic role of CD105- and CD31-assessed microvessel density (MVD) in paired biopsies and surgical specimens and the association and discrepancy between CD105- and CD31-assessed MVD in biopsies and surgical specimens. CD105- and CD31-assessed MVD was analyzed in paired biopsies and surgical specimens of 45 consecutive cases of LSCC. In the LSCC biopsies and surgical specimens, median CD105-assessed MVD was significantly higher in N+ than in N0 cases (*p* = 0.0008, and *p* = 0.0002, respectively). Disease-free survival (DFS) was associated with CD105- and CD31-assessed MVD in both biopsies and surgical specimens (*p* < 0.0001 for all specimens). Multivariable Cox’s regression showed that pathological grade (*p* < 0.0001) and CD105-assessed MVD in LSCC biopsies (*p* = 0.0209) predicted DFS. Lin’s concordance coefficient showed that CD31 overestimated MVD compared with CD105 in LSCC biopsies and surgical specimens. CD105-assessed MVD should be further investigated in larger LSCC series as a potential prognostic marker for identifying: patients at higher risk of recurrence who might warrant more aggressive therapy; and cN0 patients requiring elective neck dissection for a significant risk of regional metastasis.

## 1. Introduction

Despite advances in diagnostics and treatments, the prognosis for laryngeal squamous cell carcinoma (LSCC) has seen little improvement in the last three decades, with a 5-year survival rate of 60.9% [1]. A thorough understanding of the molecular mechanisms behind LSCC is imperative to improve patient survival, particularly in cases of advanced disease. 

Angiogenesis is a process which is fundamental to tumor growth. Several signals capable of tripping the “angiogenic switch” have been identified, including gene mutations involved in activating oncogenes and deleting tumor suppressor genes, as well as oxidative and mechanical stress and hypoxia [2,3]. Evidence of the stimulation of pro-angiogenic molecules can be seen in tumor cells and in other cells in the tumor microenvironment [3]. Metastasis occurs when angiogenic factors in the tumor micro-environment are overexpressed, facilitating the detachment of tumor cells and their migration via blood and lymph vessels [3]. Epidermal growth factor receptor (EGFR), vascular endothelial growth factor (VEGF), vascular endothelial growth factor receptor 2, endoglin (CD105), angiogenin, hypoxia-inducible factor 1, angiopoietin, inducible nitric oxide synthetase, cytochrome C oxidase subunit II, aquaporin 1, galectin 1, galectin 3, and galectin 8 have all been investigated as angiogenic markers potentially relevant to head and neck carcinoma prognosis [2,4,5,6]. The clinical and prognostic role of microvessel density (MVD) has been investigated specifically in LSCC using CD31, CD34, and CD105 as endothelial targets [2,7]. CD105 is also active in angiogenesis [8,9].

CD105 is a homodimeric transmembrane glycoprotein, and an auxiliary receptor of transforming growth factor-β (TGF-β), which binds to TGF-β1 and TGF-β3. It modulates TGF-β signaling by interacting with type I and type II TGF-β receptors, and activates a complex signaling pathway involving endothelial cell proliferation, migration and adhesion [10,11,12]. A recent meta-analysis on 30 studies involving 3,613 cancer patients found strong evidence of an increased CD105 expression in tumor microvessels correlating with poor overall survival, disease-free survival (DFS), and cancer-specific survival rates [10].

Several tissue biomarkers have been investigated for their prognostic role in LSCC [13], mostly using material obtained from surgical specimens. The question of whether information obtainable from studies on surgical specimens could be gleaned in advance by analyzing pretreatment biopsies is still unanswered. With this possibility in mind, the larynx as a site of disease could be particularly challenging because biopsies obtained by microlaryngoscopy are extremely small. The well-known heterogeneity of LSCC [14] also raises concerns about the accuracy of immunohistochemical findings in such small biopsy specimens.

The main aims of the present study were: (i) to examine the prognostic role of CD105- and CD31-assessed MVD in paired biopsy and surgical specimens of LSCC, based on univariable and multivariable statistical analyses; and (ii) to investigate the association and discrepancy between CD105- and CD31-assessed MVD in such biopsies and surgical specimens.

## 2. Results

### 2.1. Conventional Clinical-Pathological Variables and Prognosis

Thirty-one patients (68.9%) experienced no disease recurrence after treatment, while 14 (31.1%) relapsed after a median of 11 months (range 4–24 months). Univariable Cox’s proportional hazards model identified pathological grade (*p* = 0.030) and N status (with a trend towards significance *p* = 0.055) as predictive of DFS. Table 1 shows the association between the other conventional pathological variables and DFS.

### 2.2. Association between Clinical-Pathological Variables and Neo-Angiogenesis Marker (CD105 and CD31) Expression in Biopsies and Surgical Specimens

In the biopsies (Table 2), the median CD105-assessed MVD was significantly higher in N+ than in N0 patients (*p* = 0.0008). The median CD31-assessed MVD was also significantly higher in the biopsies of N+ patients (*p* = 0.0025). In the surgical specimens too (Table 2), the median CD105- and CD31-assessed MVD values were both significantly higher in N+ than in N0 patients (*p* = 0.0002 and *p* = 0.0019, respectively).

**Figure 1:**
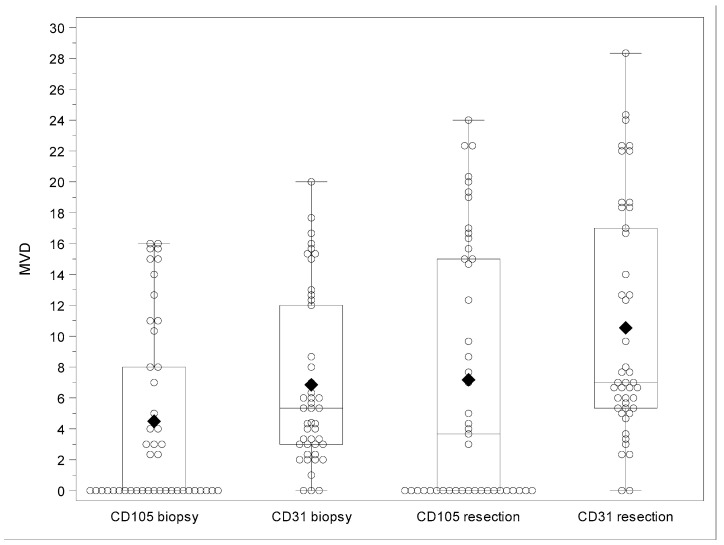
Box- and scatter-plot of CD105- and CD31-associated MVD in biopsies and surgical specimens. The bottom and top edges of the box indicate the interquartile range (IQR). The diamond inside the box indicates the mean value and the line inside the median value. The whiskers extending from each box indicate the range of values that are outside of the interquartile range corresponding to a distance less than or equal to 1.5 × IQR or the minimum and maximum value in case outliers are not present.

**Figure 2:**
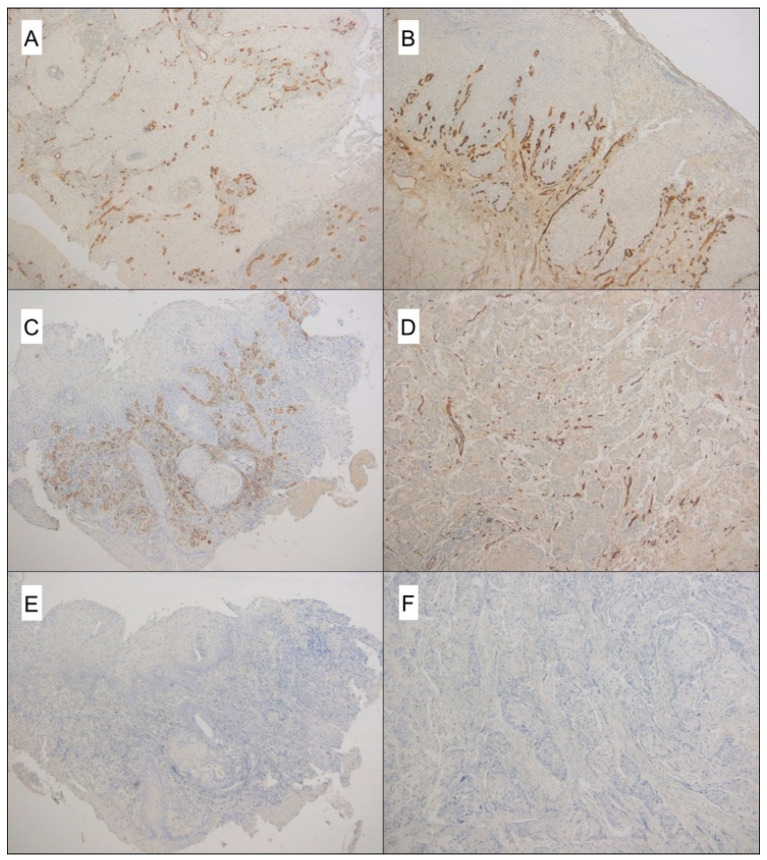
CD105- and CD31-assessed MVD in both biopsies and surgical specimens. (**A**) CD105 reveals a large number of microvessels in this LSCC biopsy (original magnification 50×); (**B**) the paired surgical specimen shows a large number of CD105-positive vessels (original magnification 50×); (**C**) a high MVD assessed by CD31 immunostaining in a LSCC biopsy (original magnification 50×); (**D**) same case, showing CD31-positive microvessels in the paired surgical specimen (original magnification 50×); (**E**) negative control of the LSCC biopsy (same cases as C); (**F**) negative control of its paired surgical specimen. In E and F, the primary antibody was replaced by phosphate buffer saline (original magnification 50×).

Figure 1 summarizes the distribution of CD105- and CD3- assessed MVD values on biopsies and surgical specimens, and Figure 2A–F show examples of high CD105- and CD31-assessed MVD in both biopsies and surgical specimens.

### 2.3. CD105- and CD31-assessed MVD in Biopsies and Paired Surgical Specimens, and LSCC Prognosis

**Figure 3:**
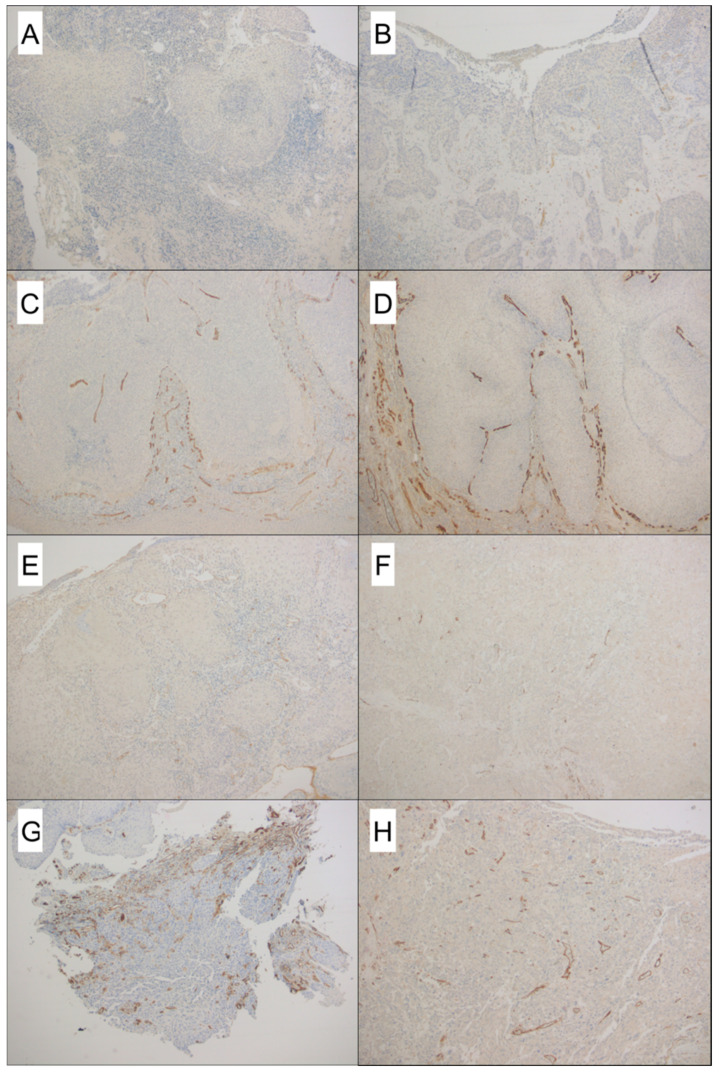
A case that did not recur, showing nearly absent CD105 staining in pre-operative biopsy (**A**) and few microvessels in surgical specimen (**B**). On the contrary, (**C**,**D**) highlight a high CD105-MVD count on both biopsy and paired surgical specimen, respectively, in a patient who developed early recurrence. CD31-assessed MVD in a patient who did not develop recurrence: biopsy (**E**) and surgical specimen (**F**). High CD31-MVD in both biopsy (**G**) and paired surgical specimen (**H**) in a case who developed local disease relapse (Original magnification 50× **A**–**H**).

**Figure 4:**
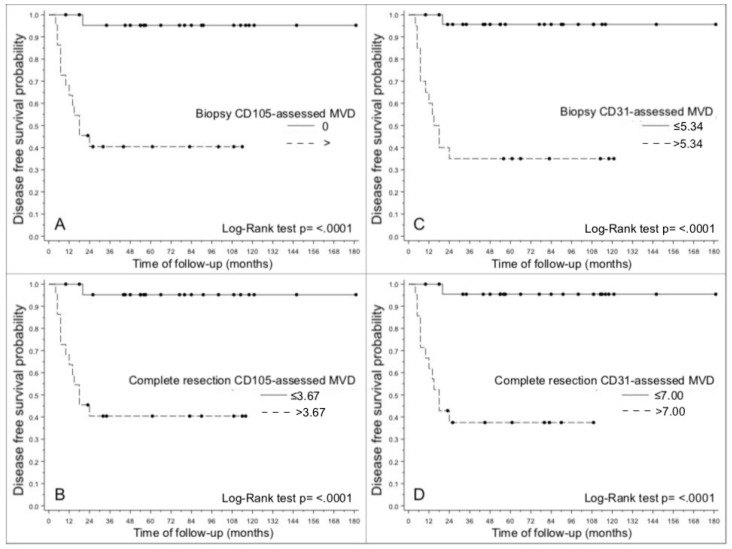
Kaplan-Meier graphs showing differences in disease-free survival: (**A**) between cases with CD105-assessed MVD > 0 vs. MVD = 0 in LSCC biopsies; (**B**) between cases with CD105-assessed MVD > 3.67 vs. MVD ≤ 3.67 in surgical specimens; (**C**) between cases with CD31-assessed MVD > 5.34 vs. MVD ≤ 5.34 in LSCC biopsies; (**D**) between cases with CD31-assessed MVD > 7 vs. MVD ≤ 7 in surgical specimens.

DFS was associated with CD105- and CD31-assessed MVD (Figure 3A–H) in both biopsies and surgical specimens (*p* < 0.0001 for all specimens) (Table 1). DFS was lower in patients with a CD105-assessed MVD in biopsies >0 (*p* < 0.0001) (Figure 4A), and in patients with a CD105-assessed MVD in surgical specimens >3.67 (*p* < 0.0001) (Figure 4B). Analyzing the more conventional pan-endothelial antigen CD31, DFS was lower in patients with both a CD31-assessed MVD > 5.34 in biopsies (*p* < 0.0001) (Figure 4C), and a CD31-assessed MVD >7 in surgical specimens (*p* < 0.0001) (Figure 4D).

Table 3 summarizes the accuracy of the statistical model (Uno’s concordance statistic)—based on C105- and CD31-assessed MVD in biopsies and surgical specimens—in predicting DFS, in terms of the agreement between observed and predicted outcomes. The accuracy was comparable for biopsies surgical specimens, since all 95% confidence intervals overlapped (despite a better performance for the surgical specimens). 

The multivariable Cox’s regression (considering N-status, pathological grading, CD105-assessed MVD in biopsies) found that both pathological grade (*p* < 0.0001; G3 versus G1–G2, HR = 1.241, 95%CI 1.128–1.365) and CD105-assessed MVD in biopsies (*p* = 0.0209; HR = 3.913 95%CI 1.229–12.453) predicted DFS after primary treatment for LSCC.

### 2.4. Comparison between CD105- and CD31-Assessed MVD in Biopsies and Paired Surgical Specimens of LSCC

**Figure 5:**
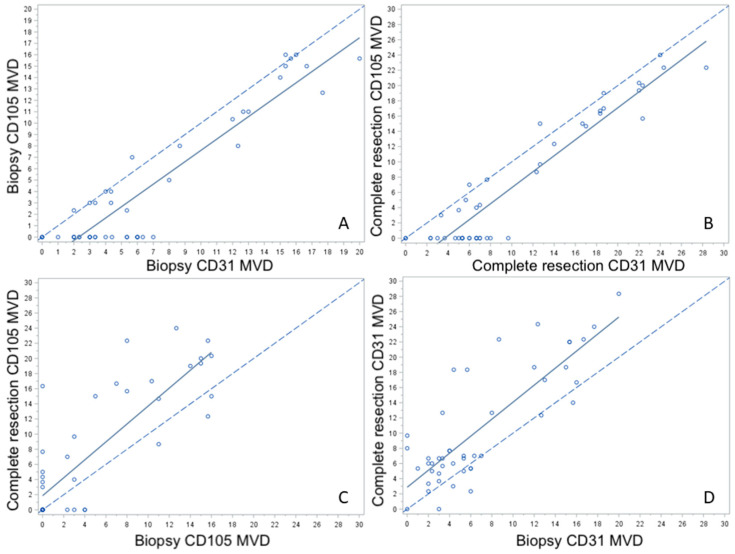
Scatter plots showing CD105- and CD31-assessed MVD in paired LSCC biopsies and surgical specimens (**A**–**D**).

Figure 5 and Table 4 illustrate graphically and numerically, respectively, the agreement between CD31 and CD105 and between biopsies and resection specimens. CD31 and CD105 graphs show a shift for both biopsy and resection specimens denoting a constant upward bias reflected also by a sub-optimal concordance correlation coefficient (0.8519 and 0.8593, respectively; Figure 5A,B). Biopsy and resection graphs for both CD105 and CD31 present a drift indicating an ever-increasing bias and a modest concordance correlation coefficient (0.7352 and 0.6775, respectively; Figure 5C,D). 

## 3. Discussion

Biological markers analysis on biopsies may potentially provide relevant prognostic data to support clinical decision making. Since during biopsy only a small part of a solid tumor is removed, it is not necessarily representative of the entire neoplasm. Considering limited available data, it is still doubtful whether the results of immunohistochemistry performed on head and neck cancer biopsy material in the diagnostic phase can be used in clinical decision making; well-known head neck carcinoma heterogeneity [14] raises the concern that immunohistochemistry methods may not be accurate on small biopsy specimens. The very small size of the biopsy material is often a critical aspect in pathological diagnosis, in particular of laryngeal malignancies [15,16]. In addition, the pattern of expression may differ between the proteins. Takes et al. [14] compared the expression of p53, Rb protein, E-Cadherin, Ep-CAM, desmoplakin 1, and cortactin in biopsy and resection material of 26 LSCCs. A variable rate of mismatches between the scoring on biopsy and resection material was found for the different proteins. In oropharyngeal SCC, Brcic et al. [17] found a high correlation of p16 expression evaluation nevertheless some cases might be misclassified on a small biopsy. Moreover, they demonstrated high correlation of PD-L1 expression between resections and small biopsies. 

As extensively reported in several previous studies, we identified a statistically significant correlation between N status and DFS. In our series, pathological grade also predicted DFS in both univariable and multivariable analyses. In the present study, besides confirming the role of CD105 expression in LSCC surgical specimens in predicting worse prognosis [4], and a higher risk of nodal metastases [18], the results of our univariable analysis reveal for the first time that CD105- and CD31-assessed MVD in LSCC biopsies has prognostic potential too (Figure 4A,C, respectively). CD105-assessed MVD in biopsies remained independently prognostic for DFS on multivariable analysis as well. In terms of the agreement between the observed and predicted outcomes, the accuracy of CD105- and CD31-assessed MVD in biopsies in predicting DFS was good (0.8461 and 0.8283, respectively) [19]. It was also comparable for the two markers, since all the 95% confidence intervals overlapped, with a slightly better performance in surgical specimens (see Table 3). When CD105- and CD31-assessed MVD were compared in both biopsies and surgical specimens, there was a shift in the location of the regression line vis-à-vis the bisector line. This indicated that using CD31 led to an overestimation of MVD with respect to CD105. In fact, CD31 was often expressed in tumor microvessels in samples showing no CD105 staining (see also Figure 5A,B). These findings can be explained by the previously reported evidence [20] of CD105 being preferentially expressed on active endothelial cells during the neo-angiogenic process, whereas pan-endothelial antigens such as CD31 are also expressed by stable vessels trapped inside the tumor. This would suggest a greater specificity of CD105 than CD31 for assessing the tumor neo-vasculature [21,22], though CD31-assessed MVD performed well enough as a marker for it to still be useful, also due to its wider diffusion and better standardization.

To the best of our knowledge, this study was the first to examine the prognostic roles of CD105- and CD31-assessed MVD in paired biopsies and surgical specimens of LSCC, and the associations and discrepancies between them. The main strength of our investigation lies in the homogeneity of the patient population since: (i) all patients underwent surgery; (ii) their surgical treatment was performed consecutively by the same team; (iii) only squamous cell carcinomas located in the larynx were considered; (iv) for each case, both biopsies and surgical specimens were assessed and compared; (v) both CD105 and CD31 were used to assess MVD; (vi) clinical-radiological follow-up criteria were defined; (vii) a tailored multivariable statistical model was used to analyze the variables potentially predictive of DFS. In addition, over the course of about 15 years [23], the pathologists in our research group have gained plenty of experience of assessing MVD from the immunohistochemical expression of CD105 in head and neck malignancies [24], also in association with oncogenes [25], or tumor suppressors [26]. The main weaknesses of our study concern the retrospective setting and the limited number of cases considered.

The optimal management of locally advanced LSCC needs to rely not only on conventional clinical-pathological factors, but also on molecular biomarkers, based on the concept of personalized medicine. To make the most of prognostic biomarkers, sound data on their expression should be obtainable in the diagnostic phase. For instance, if a protein’s expression can identify patients who would benefit from elective treatment of the neck, it would be crucial to establish this from biopsies. In the present exploratory study, CD105-assessed MVD in LSCC biopsies was strongly associated with N status (see Table 2). Importantly, CD105 expression in activated endothelial cells—even in biopsies—could identify patients at higher risk of disease recurrence who might benefit from a more aggressive therapy, including postoperative radiotherapy or chemo-radiotherapy. It is important to bear in mind that, in addition to conventional combined treatments for LSCC, anti-angiogenic therapy is one of the most promising strategies for this cancer’s treatment. Inhibiting CD105 should impede signaling by TGFβ, potentially shutting off an escape pathway during VEGF blockade [27]. Clinical trials have recently begun on a chimeric IgG1 anti-CD105 monoclonal antibody (TRC105) that binds human endoglin with high avidity, indicating that CD105 might be a promising anti-angiogenic target of cancer therapy [10]. It inhibits angiogenesis and tumor growth through endothelial cell growth inhibition, antibody-dependent cellular cytotoxicity, inhibition of signal transduction and/or aberrant signal transduction, induction of apoptosis, and complementing VEGF inhibitors [28,29,30]. In describing a novel mechanism by which CD105 initiated and regulated VEGF-driven angiogenesis, Tian et al. [31] recently provided a rationale for combining anti-VEGF and anti-CD105 therapy in patients with cancer.

## 4. Materials and Methods

### 4.1. Patients

The study was conducted in accordance with the principles of the Helsinki Declaration. The data of this retrospective investigation were examined in compliance with Italian privacy and sensitive data laws and the in-house rules of Padova University’s Otolaryngology Section. All patients preoperatively signed a consent form for disclosure of privacy in managing personal data for scientific purposes. In particular, they consented “to the use of their clinical data for scientific research purposes in the medical, biomedical and epidemiological fields, also in order to be recalled in the future for follow-up needs”. Before undergoing surgery, all patients included in the study signed a detailed informed consent form.

Our investigation involved 45 consecutive patients (41 males, 4 females; mean age 65.2 ± 8.1 years) with early and locally advanced primary LSCC who underwent partial or total laryngectomy at the Otolaryngology Section of Padova University less than thirty days after biopsy diagnosis between 1999 and 2013. None of the patients had undergone previous treatments for laryngeal cancer. Unilateral or bilateral cervical lymph node dissection was performed in 42 cases. As previously reported [32,33], all patients had undergone microlaryngoscopy with laryngeal biopsy at our institution, upper aerodigestive tract endoscopy, neck ultrasonography (with or without fine needle aspiration cytology), head and neck contrast-enhanced computerized tomography (CT) and/or magnetic resonance imaging, chest X-ray, and liver ultrasonography. Pathological findings warranted postoperative radiotherapy (with or without concomitant chemotherapy) according to current guidelines in 21 cases. Table 1 provides details of patients’ clinical-pathological features, based on the 8th edition of the TNM Classification of Malignant Tumors [34]. No distant metastases (M) were detected at diagnosis. As explained elsewhere [35,36], the clinical follow-up after treatment at our institution (adjustable to patient’s individual characteristics) was scheduled as follows: (i) once a month for the 1st year; (ii) every 2 months in the 2nd year; (iii) every 3 months in the 3rd year; (iv) every 4 months in the 4th year; (v) every 6 months in the 5th year; and (vi) every 12 months thereafter. Neck ultrasonography and chest X-rays were performed at least yearly. Contrast-enhanced CT of the neck, total body positron emission tomography, chest CT, neck and liver ultrasonography were repeated if clinically indicated. The median follow-up was 61 months (range 10–181 months).

### 4.2. Immunohistochemistry

Immunohistochemistry was performed on formalin-fixed and paraffin-embedded sections, 2.5 μm thick, obtained from each pair of LSCC preoperative biopsies and surgical specimens. An automated system (Benchmark-Ultra, Ventana, Tucson, AZ, USA) was preferred for immunohistochemical analyses. The primary antibodies used were: CD105 (Mouse Monoclonal, clone SN6, dilution 1:100, Santa Cruz Biotechnology, Inc. Santa Cruz, CA, USA) and CD31 (Mouse Monoclonal, clone JC70A, dilution 1:50, Dako, Glostrup, Denmark). Although with significantly different aims, CD105 and CD31 expressions were previously evaluated in LSCC specimens [2].

The sections were dehydrated, cleared, mounted and counterstained with Meyer’s haematoxylin. Appropriate positive controls were run concurrently, according to manufacturer’s protocols. The primary antibody was replaced by phosphate buffer saline as a negative control. 

Slides stained for CD105 and CD31 were examined to ascertain MVD in the tissue samples, considering intra-tumoral and peri-tumoral areas (<500 µm within the tumor margin). Each slide was initially examined by a dedicated head and neck pathologist (L.A.) under the light microscope at 100× magnification, identifying three areas with the largest number of stained vessels, defined as “hot spots” [37]. Sections without at least three “hot spots” identifiable (especially small biopsy samples) were rejected for the purposes of the present study. Vessels in each “hot spot” were counted by the pathologist under the light microscope at 400× magnification (high-power fields (HPF), with 0.237 mm^2^/field under the light microscope). MVD was calculated as the arithmetical mean of the number of microvessels counted.

The microvessel count considered all positively stained round, oval or irregular structures that were separate from other profiles or connective tissue elements, and from each other. Vessels with a muscle layer were excluded, as were areas of necrosis.

### 4.3. Statistical Analysis

The statistical analyses were performed with SAS 9.4 for Windows (SAS Institute Inc., Cary, NC, USA). The data are presented as means and standard deviations, medians and interquartile ranges (IQRs) for quantitative variables, and as counts and percentages for the categorical variables. The association between the CD105- and CD31-assessed MVD in biopsies and surgical specimens and the dichotomized clinical-pathological features was assessed using Wilcoxon’s rank sum test.

For both types of specimen, the accuracy with which CD105- and CD31-assessed MVD predicted time to LSCC recurrence was tested using a univariable Cox’s regression analysis, and expressed as Uno’s concordance statistic. The results of the Cox’s regression are expressed as *p*-values, hazard ratios and 95% confidence intervals. Uno’s concordance standard error [38] was estimated with 100 perturbation resampling, and the 95% confidence interval was calculated considering the normal approximation. The time to LSCC recurrence was calculated as the time from completing treatment for the primary tumor to LSCC recurrence, or to last follow-up for patients experiencing no recurrence.

The DFS predicted from the CD105- and CD31-assessed MVD for both types of specimen was represented graphically with Kaplan-Meier curves, classifying MVD by median value (≤/>), and assessed with a log-rank test. Clinical-pathological predictors and the novel neo-angiogenesis markers found statistically significant in the biopsies at the 0.10 level in univariable Cox’s regression analyses were input in a multivariable regression analysis with backward selection, considering the 0.05 cut-off for statistical significance. Clinical-pathological predictors collinear (associated) with the neo-angiogenesis marker were disregarded. The proportionality assumption of the Cox’s models was checked with a Kolmogorov-type supremum test using 1,000 resampling. Agreement between CD105- and CD31-assessed MVD for the two types of specimen was examined with Lin’s concordance coefficient [39] and the 95% confidence interval was obtained with 2,000 bootstrap resampling.

A *p*-value < 0.05 was considered indicative of statistical significance.

## 5. Conclusions

Based on the results of the present study, a high CD105-assessed MVD is worth further investigating in larger series of LSCC biopsies as a prognostic marker potentially useful for identifying: (i) patients at higher risk of recurrence, who might warrant more aggressive therapy; and (ii) cN0 patients requiring elective neck dissection due to a significant risk of occult regional metastases. 

## Figures and Tables

**Table 1:** Descriptive statistics of CD105- and CD31-assessed microvessel density (MVD) (biopsies and surgical specimens) and of conventional clinical-pathological features according to laryngeal squamous cell carcinoma (LSCC) recurrence, and univariable Cox regression analysis results for LSCC recurrence.

No. = 45	Without LSCC Recurrence (No. = 31)	With LSCC Recurrence (No. = 14)	* *p*-Value	HR (95%CI)
**CD105 MVD (biopsy)**				
Mean (SD)	1.56 (3.47)	10.98 (4.86)		
Median (IQR)	0.00 (0.00–2.34)	11.84 (8.00–15.00)	<0.0001	1.221 (1.118; 1.334)
**CD31 MVD (biopsy)**				
Mean (SD)	4.36 (3.85)	12.36 (4.65)		
Median (IQR)	3.34 (2.34–5.34)	13.84 (8.67–15.67)	<0.0001	1.202 (1.107; 1.306)
**CD105 MVD (surgical specimen)**				
Mean (SD)	3.05 (5.68)	16.31 (5.53)		
Median (IQR)	0.00 (0.00–4.34)	16.84 (15.00–20.00)	<0.0001	1.195 (1.104;1.294)
**CD31 MVD (surgical specimen)**				
Mean (SD)	7.19 (5.54)	17.98 (5.82)		
Median (IQR)	6.00 (5.00–7.67)	18.67 (14.00–22.34)	<0.0001	1.153 (1.083; 1.228)
**pT**				
T1–T2	17 (54.8%)	04 (28.6%)		
T3–T4	14 (45.2%)	10 (71.4%)	0.1304	2.450 (0.767; 7.823)
**Pathological grade**				
G1–G2	23 (74.2%)	06 (42.9%)		
G3	08 (25.8%)	08 (57.1%)	0.0302	3.250 (1.119; 9.433)
**N status**				
N0 **	26 (83.9%)	08 (57.1%)		
N+	05 (16.1%)	06 (42.9%)	0.0553	2.832 (0.977; 8.212)
**Tumor stage**				
I–II	15 (48.4%)	04 (28.6%)		
III–IV	16 (51.6%)	10 (71.4%)	0.2510	1.973 (0.618; 6.298)

* *p*-value: Cox’s proportional hazards model; ** N0 status: cN0 3 cases; pN0 31 cases. MVD: microvessel density; SD: standard deviation; IQR: interquartile range; HR: hazard ratio; 95% CI: 95%.

**Table 2:** CD105- and CD31-assessed MVD in biopsies and surgical specimens vis-à-vis conventional clinical-pathological variables.

Variable	No. of Cases	CD105-Assessed MVD (biopsy) Mean (SD) Median (IQR)		CD105-Assessed MVD (Surgical Specimen) Mean (SD) Median (IQR)		CD31-Assessed MVD (Biopsy) Mean (SD) Median (IQR)		CD31-Assessed MVD (Surgical Specimen) Mean (SD) Median (IQR)	
pT1–2	21	3.16 (4.99) 0.00 (0.00–4.00)	*p* = 0.1296	5.87 (8.24) 0.00 (0.00–14.67)	*p* = 0.2542	6.26 (4.77) 4.40 (3.00-7.00)	*p* = 0.9364	9.27 (7.68) 7.00 (4.67–17.00)	*p* = 0.2318
pT3–4	24	5.65 (6.45) 3.00 (0.00-12.50)		8.32 (8.43) 6.00 (0.00–15.84)		7.36 (6.16) 5.51 (2.17–13.84)		11.66 (7.34) 8.84 (6.00–17.51)	
N0 *	34	2.67 (4.30) 0.0 (0.00–4.00)	*p* = 0.0008	4.53 (7.45) 0.00 (0.00–7.00)	*p* = 0.0002	5.26 (4.41) 4.17 (2.34–6.00)	*p* = 0.0025	8.42 (6.74) 6.67 (5.00–8.00)	*p* = 0.0019
pN+	11	10.12 (6.72) 14.00 (3.00-15.67)		15.37 (5.03) 15.00 (12.34–19.34)		11.77 (5.91) 15.00 (4.40–15.67)		17.09 (5.98) 17.00 (12.67–22.00)	
Stage I–II	19	2.91 (4.86) 0.00 (0.00–4.00)	*p* = 0.1112	4.86 (8.00) 0.00 (0.00–7.67)	*p* = 0.0920	6.00 (4.75) 4.34 (3.00–7.00)	*p* = 0.7042	8.39 (7.54) 6.67 (3.67–7.67)	*p* = 0.0804
Stage III–IV	26	5.64 (6.38) 3.00 (0.00–11.00)		8.87 (8.32) 7.84 (0.00–16.34)		7.47 (6.05) 5.51 (2.34–13.00)		12.12 (7.23) 11.01 (6.00–18.34)	
Grade 1–2	29	3.76 (5.78) 0.00 (0.00–4.00)	*p* = 0.1536	5.85 (7.94) 0.00 (0.00–9.67)	*p* = 0.2020	6.41 (5.76) 4.34 (3.00–7.00)	*p* = 0.3363	9.17 (7.28) 6.67 (5.00–12.67)	*p* = 0.1318
Grade 3	16	5.81 (6.02) 4.50 (0.00–9.50)		9.59 (8.75) 11.84 (0.00–16.51)		7.65 (5.16) 6.00 (3.50–12.51)		13.03 (7.51) 12.51 (6.01–20.17)	

* cN0: 3 cases; pN0: 31 cases, MVD: microvessel density; SD: standard deviation; IQR: interquartile range.

**Table 3:** Accuracy of statistical model based on each listed variable in predicting recurrence-free survival, in terms of agreement between observed and predicted outcomes (Uno’s concordance statistic).

Variable	UNO’s Concordance Statistic	SE *	95%CI
CD105-assessed MVD (biopsy)	0.8461	0.0396	0.7685; 0.9237
CD31-assessed MVD (biopsy)	0.8283	0.0444	0.7413; 0.9153
CD105-assessed MVD (surgical specimen)	0.8880	0.0316	0.8261; 0.9499
CD31-assessed MVD (surgical specimen)	0.8520	0.0442	0.7654; 0.9386

MVD: microvessel density; SE: standard error: 95% confidence interval. * SE obtained with 100 perturbation resamplings.

**Table 4:** Agreement between CD105- and CD31-assessed MVD in LSCC biopsies and surgical specimens.

Variables Tested for Concordance	Concordance Coefficient *	Bootstrap 95% CI **
CD105-assessed MVD(biopsy)/CD31-assessed MVD(biopsy)	0.8519	0.7589; 0.9120
CD105-assessed MVD(surgical specimen)/CD31-assessed MVD(surgical specimen)	0.8593	0.7805; 0.9082
CD105-assessed MVD(biopsy)/CD105-assessed MVD (surgical specimen)	0.7352	0.565; 0.8329
CD31-assessed MVD(biopsy)/CD31-assessed MVD (surgical specimen)	0.6775	0.5273; 0.7865

MVD: microvessel density, * Lin’s concordance coefficient, ** 95% bootstrap confidence interval obtained with 2,000 samplings.
